# Association of Depressive Symptom Scores with Multimodal Brain Imaging and Behavioral Phenotypes: A Resting-State, Task-FMRI, and Clinical Comorbidity Study Based on the Human Connectome Project

**DOI:** 10.3390/brainsci16080884

**Published:** 2026-08-19

**Authors:** Fufeng Zheng, Song Zhang, Xiaoying Tang, Guangfei Li

**Affiliations:** 1 Department of Intelligent Medicine and Imaging Technology, School of Medical Science and Engineering, Beijing Institute of Technology, Beijing 100081, China; 3120225719@bit.edu.cn; 2 Beijing Yes Medical Devices Co., Ltd., Beijing 101111, China; 3 Department of Biomedical Engineering, College of Chemistry and Life Science, Beijing University of Technology, Beijing 100124, China

**Keywords:** depression symptoms, rsFC, social cognition, fMRI

## Abstract

**Objective:** Depressive symptoms exist on a continuum in the general population, yet the underlying neurobiological mechanisms, particularly the interplay between resting-state networks and task-evoked social cognitive responses, remain elusive. **Methods:** Leveraging the Human Connectome Project (HCP) dataset, we included 867 participants. With depression scores as the independent variable and age/sex as covariates, we systematically examined associations with sleep quality, negative emotions, sensory scores, gray matter volume (GMV), fractional amplitude of low-frequency fluctuations (fALFF), multi-seed resting-state functional connectivity (rsFC), as well as brain activation and behavioral performance during working memory, emotion recognition, social cognition, relational reasoning, language comprehension, and gambling tasks. The statistical threshold was set at voxel-level *p* < 0.001 (uncorrected) combined with cluster-level FWE correction at *p* < 0.05. **Results:** (1) Depression scores were positively correlated with sleep disturbances, negative emotions (anger/fear), and pain. (2) In resting-state, depression scores negatively correlated with ventral striatum (VS)–cerebellum/parahippocampal gyrus/fusiform rsFC, yet positively correlated with pregenual anterior cingulate cortex (preACC)–supplementary motor area (SMA) rsFC. (3) In task-fMRI, only the social task showed a positive association with task accuracy and regional activation in bilateral pre/postcentral gyri, superior temporal gyri, left middle frontal gyrus, and SMA/paracentral lobule. **Conclusions:** Elevated depression scores are linked to a pattern that may reflect relative decoupling between reward and perceptual systems, along with enhanced connectivity in cognitive control circuits. Socially, high scorers exhibit a pattern suggestive of compensatory hypervigilance, accompanied by enhanced behavioral performance. This study provides multidimensional evidence for the dimensional neural representation of depressive symptoms.

## 1. Introduction

### 1.1. Background

Major depressive disorder (MDD) is one of the most prevalent psychiatric disorders worldwide, with a lifetime prevalence of approximately 6.4–10.4% [1]. Depression has been conceptualized as a categorical diagnostic entity—individuals either meet the Diagnostic and Statistical Manual of Mental Disorders (DSM) criteria or they do not. However, accumulating evidence suggests that depressive symptoms are continuously distributed in the general population, and even subclinical depressive symptoms below the diagnostic threshold are associated with significant functional impairment and reduced quality of life [2,3,4]. The neurobiological underpinnings of depression may vary gradually along the continuum of symptom severity, rather than exhibiting “all-or-none” brain network abnormalities only in the disease state [5]. Therefore, examining the correlation between depression scores and neuroimaging metrics in healthy populations can help reveal early neurobiological markers of depressive symptoms, free from confounding factors such as medication and disease course, thereby providing a unique dimensional perspective for understanding the pathogenesis of depression.

### 1.2. Resting-State Functional Connectivity: The Brain Network Basis of Depression

Resting-state functional magnetic resonance imaging (rs-fMRI) measures the temporal synchronization of low-frequency blood oxygen level-dependent signals in the absence of explicit tasks, reflecting intrinsic functional connectivity between different brain regions [6,7,8,9]. In recent years, functional connectomics has provided a quantitative theoretical framework and analytical tools for parsing variations in the organization and function of brain networks in depression [10,11]. A large-scale meta-analysis exhibited significant alterations in resting-state network connectivity (rsFC), including both increased and decreased connectivity within the default mode network (DMN), increased connectivity in the frontoparietal network (FPN), and increased cross-network connectivity between DMN-FPN and limbic network-DMN; however, the direction and regional specificity of resting-state network disruptions remain highly inconsistent across the existing literature [10]. The review by Sun et al. similarly emphasized the substantial heterogeneity in findings from rsFC studies of depression [12]. This inconsistency may stem from clinical heterogeneity (e.g., depression subtype differences), insufficient sample sizes, and variability in analytical methods.

In non-clinical populations with subclinical depressive symptoms, structural alterations have been reported in the hippocampus, anterior cingulate cortex, and anterior insula—regions similarly affected in MDD [13]. A multimodal meta-analysis of subthreshold depression found spatially convergent structural and functional abnormalities primarily in the left insula, along with structural atrophy and decreased functional activity in the right pallidum and thalamus [14]. For spontaneous brain activity, alterations in the fractional amplitude of low-frequency fluctuations (fALFF) have been documented in MDD across frontal–parietal regions [15], but whether fALFF changes are detectable along the dimensional continuum of depressive symptoms in non-clinical populations remains largely unexplored.

### 1.3. Task-Fmri: The Act of Depression on Multiple Cognitive Abilities

Beyond resting-state abnormalities, patients with depression also exhibit altered neural responses across multiple cognitive tasks. The Human Connectome Project (HCP) database includes seven task-fMRI paradigms, and these tasks provide an opportunity to systematically evaluate the neural correlates of depression scores across different cognitive domains.

Studies have demonstrated that depression impairs processing speed, attention, executive function [16,17,18] and social perception [1]. A recent review of task-fMRI in MDD has summarized functional abnormalities across three domains: executive function, negative bias, and reward processing. Deficits in executive function manifest as inefficiency in the FPN and failure to suppress the DMN during n-back and Stroop tasks; negative bias is characterized by hyperactivity in bottom-up emotional responses and failure of top-down regulation; and reward processing abnormalities are consistently associated with blunted reward prediction error signals and hypoactivation of thalamo–striatal–prefrontal circuitry [19]. However, existing studies have primarily focused on group comparisons between clinically depressed patients and healthy controls, with less attention paid to the relationship between the continuous dimension of depressive symptoms and neural responses during cognitive tasks in non-clinical samples. Moreover, no study to date has systematically compared the sensitivity of multiple cognitive task paradigms to depression scores within the same large-scale cohort, leaving unanswered which cognitive domain is most closely tied to subclinical depressive symptomatology at the neural level.

### 1.4. The Present Study

In summary, the research gaps remain in the current literature: (1) the association patterns between depression scores and multi-seed rsFC in non-clinical samples remain unclear (seed regions chosen based on their roles in reward processing, emotion regulation, and the neurocircuitry of depression); (2) existing task-fMRI studies have rarely compared the sensitivity of different cognitive tasks to depression scores.

**Figure 1:**
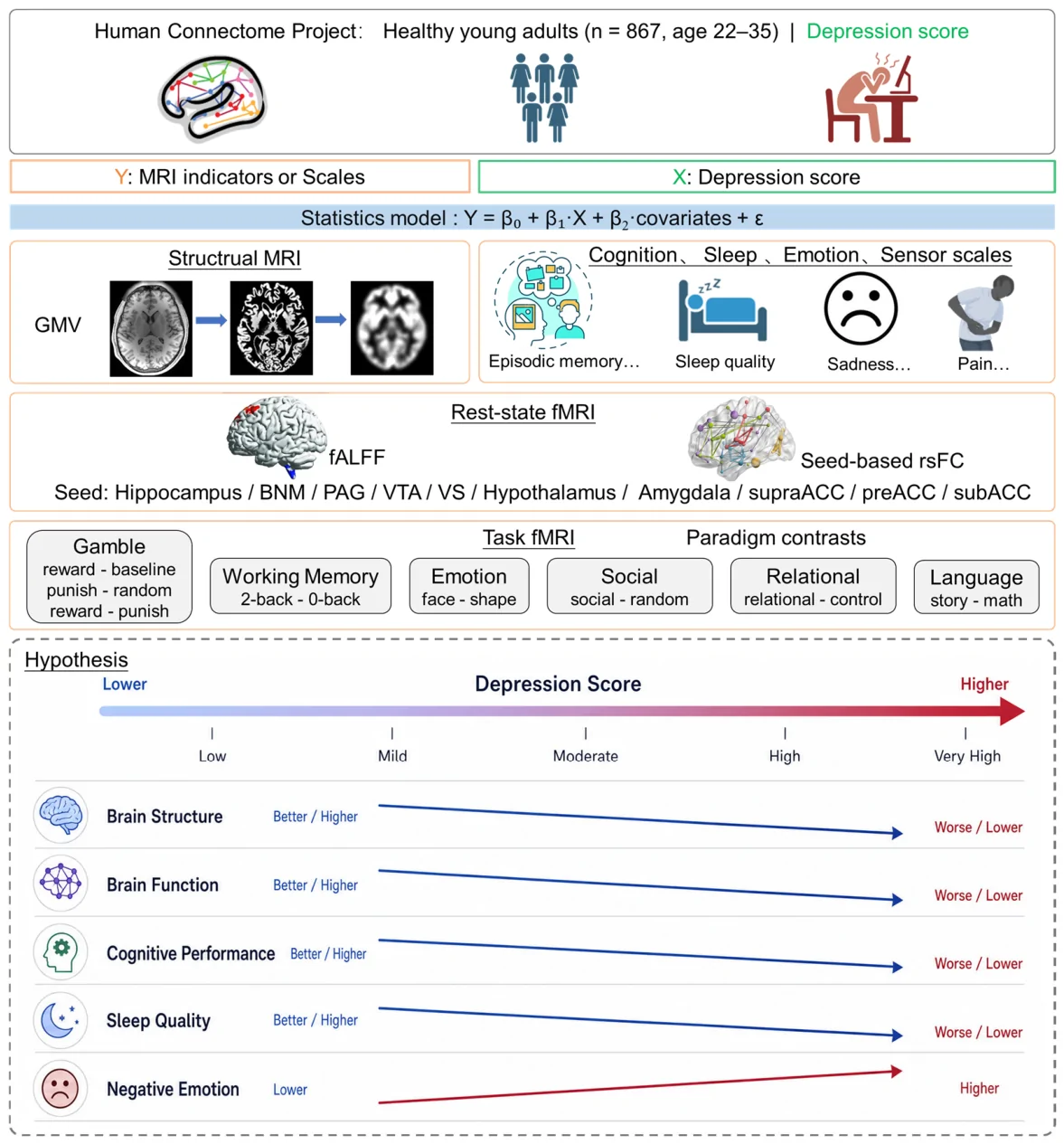
Overall research approach and hypotheses.

Several prior studies have leveraged the HCP dataset to examine depressive symptoms in relation to brain imaging measures. Cheng et al. identified functional connectivities that mediate the association between depressive problems and sleep quality using resting-state data [20]. Jackson and Bernard examined cerebellum–basal ganglia motor network integration in relation to trait depression and hyperactivity [21]. Yuan et al. explored the neural correlation between emotion recognition ability and depressive symptoms using the emotion recognition task [22]. However, these studies were limited to single modalities (resting-state only or single-task only) and focused on specific brain systems or isolated cognitive domains. The present study leverages the HCP database to systematically investigate associations between depression scores and multidimensional behavioral phenotypes (sleep, cognition, emotion, sensor), rsFC (multi-seed based), and activation patterns across several task-fMRI paradigms. We hypothesize that: (1) depression scores are positively correlated with sleep disturbances and negative emotions; (2) negatively correlated with rsFC in reward-related pathways and positively correlated with rsFC in cognitive control-related pathways; and (3) among task-fMRI paradigms, social perception may be more sensitive to depression scores than other tasks (Figure 1).

## 2. Materials and Methods

### 2.1. Dataset and Demographics

We employed the HCP 1200 Subjects Release data in the current study. A total of 1096 young adults completed a resting state fMRI scan and, after exclusion of subjects missing physiological data (*n* = 80) or clinical measures (*n* = 3), or not meeting the scrubbing criteria (*n* = 146; details in Section 2.3), 867 were retained. A total of 998 participants completed fMRI scans of the seven behavioral tasks [23] and 833 of them showed good image quality across all scans. The intersection of the samples of 867 and 833 participants resulted in a pool of 687 participants (345 men) for inclusion in this study. All subjects were physically healthy with no severe neurodevelopmental, neuropsychiatric or neurological disorders. The HCP study was approved by the Washington University Institutional Review Board (IRB #201204036).

All participants were assessed with the Achenbach Adult DSM-Oriented Scales. The DSM-Oriented depression problems subscale comprises 14 items, each scored from 0 to 2, so the total score ranges from 0 to 28, with a higher score indicating more severe depression.

Participants were evaluated with the Pittsburgh Sleep Quality Index [24], which contains 19 self-rated questions. The 19 self-rated items are combined into 7 component scores, each of which ranges from 0 to 3, with “0” and “3” each indicating no and severe difficulty with sleep. Thus, individuals range from 0 to 21 in PSQI score, with a higher score representing worse sleep quality.

Table 1 shows sex differences in demographics and clinical measures.

### 2.2. Cognition, Emotion and Sensory Measures

HCP collected cognition, emotion and sensory scales for each participant [25]. To assess future planning, a delay discounting task measured how quickly individuals devalue future money compared to immediate money, using amounts of $200 and $40,000. Sustained attention was evaluated with the Short Penn Continuous Performance Test, where participants viewed sequences of line arrangements and pressed a button when the lines formed a letter or a number. Verbal episodic memory was tested using Form A of the Penn Word Memory Test: participants were shown 20 words to remember, then later presented with 40 words (including the original 20) and asked if they had seen each word before. Fluid intelligence was measured with Form A of an abbreviated version of Raven’s Progressive Matrices. Spatial orientation was examined using the Variable Short Penn Line Orientation Test, in which participants saw two lines at different angles and had to rotate one line to become parallel with the other. Additionally, the NIH Toolbox was employed to assess episodic memory, cognitive flexibility, inhibition, vocabulary, processing speed, working memory, and reading.

To evaluate emotion recognition, the Penn Emotion Recognition Test was used. Participants viewed 40 faces and had to decide whether the facial expression shown was happy, sad, angry, scared, or neutral (no feeling). Emotional state was measured using the NIH Toolbox for the Assessment of Neurological and Behavioral Function (NIH Toolbox V.2; www.nihtoolbox.org, accessed on 9 October 2019), which included questionnaires assessing negative affect, positive affect, life satisfaction, meaning and purpose, friendship, social support, stress, and self-efficacy.

All participants were assessed for audition (words in noise), olfaction (odor identification test), pain (pain intensity and interference surveys), taste (taste intensity test), vision (EVA scores and Farnsworth test) and contrast sensitivity (Mars contrast sensitivity) using the NIH Toolbox.

### 2.3. Imaging Protocol and Preprocessing

MRI data were acquired on a customized 3T Siemens Connectome Skyra scanner (Siemens Healthcare GmbH, Erlangen, Germany) with a standard 32-channel head coil. Resting-state fMRI data were collected using a gradient-echo EPI sequence with TR = 720 ms, TE = 33.1 ms, flip angle = 52°, 2.0 mm isotropic voxels, multi-band factor = 8, and 72 slices. T1-weighted structural images were acquired using a 3D MPRAGE sequence with 0.7 mm isotropic resolution. Preprocessing followed standard HCP pipelines and included motion correction, co-registration, normalization to MNI152 space, smoothing (4 mm FWHM for fMRI; 8 mm FWHM for VBM), nuisance regression (white matter, CSF, whole-brain, and physiological signals), temporal band-pass filtering (0.009–0.08 Hz), and scrubbing for motion artifact removal (FD > 0.2 mm or DVARS > 75). For complete step-by-step details, please refer to the Supplementary Methods.

### 2.4. Structural Brain Analysis

Voxel-based morphometry (VBM) was used to compute gray matter volume (GMV) with the CAT12 toolbox (1700) (http://dbm.neuro.uni-jena.de/vbm, accessed on 8 January 2021), as detailed in the Supplementary Methods [26,27,28,29].

### 2.5. Resting State Functional Connectivity (Rsfc) and Falff

The masks of the hippocampus, basal nucleus of Meynert (BNM), periaqueductal gray (PAG), ventral tegmental area (VTA), ventral striatum (VS), hypothalamus, supragenual anterior cingulate cortex (supraACC), pregenual ACC (preACC), subgenual ACC (subACC), and amygdala were obtained from the WFU Pick Atlas (v3.0.5) (http://fmri.wfubmc.edu/software/Pickatlas, accessed on 9 October 2019) [30] or the Automated Anatomic Labeling atlas [31] and were used as the seed regions. Whole-brain voxel-wise analyses were conducted to compute the rsFCs of these seeds. The BOLD time courses of each voxel were averaged within the seed, and the correlation coefficient was computed between the time courses of the seed and all other voxels of the brain for individual participants.

Fractional amplitude of low-frequency fluctuations (fALFF) was calculated to measure spontaneous brain activity during rest. For each voxel, the time series was transformed to the frequency domain using a fast Fourier transform. fALFF was computed as the ratio of the power within the low-frequency band (0.009–0.08 Hz) to that of the entire frequency range.

### 2.6. Task Fmri Analysis

Please note that 687 participants (345 men) of the retained 867 participants were included in this study. First-level individual contrast maps were generated for working memory (2-back–0-back), emotion recognition (negative–neutral), social perception (social interaction–random motion), relational reasoning (relational–control), language comprehension (story–math), and gambling (reward–punishment, reward–baseline, punishment–baseline). For these tasks, reaction times (RT) and accuracy rate were extracted as behavioral measures.

### 2.7. Statistics

At each voxel (and for behavioral measures), a general linear model was fitted: Y = β_0_ + β_1_·depression score + β_2_·covariates + ε. In group analyses, a whole-brain regression of brain features (i.e., GMV, rsFC and contrasts of task) against depression score was conducted, with age, sex and total intracranial volume (TIV, only for GMV analysis) as covariates.

Given the multimodal and multitask nature of this study, all analyses were treated as exploratory and hypothesis-generating. Whole-brain imaging results were evaluated using a voxel-level threshold of *p* < 0.001 (uncorrected), combined with cluster-level FWE correction at *p* < 0.05 within each individual brain map. This within-map correction did not control for comparisons across seeds, behavioral outcomes, task contrasts, or imaging modalities. We did not apply a single unified correction across these analytical families; accordingly, all reported findings require independent replication.

Sensitivity analyses were employed to address potential non-independence due to familial relatedness (twins and siblings) as well as to assess the impact of additional covariates (BMI, education, race, and substance use).

### 2.8. Generative Artificial Intelligence (GenAI) Disclosure

In preparing the hypothesis schematic presented in Figure 1, we used the generative AI tool Lovart (1.0.20) to create the initial layout and visual components of the conceptual framework. The AI-generated draft served as a starting point; all resulting graphical elements were carefully reviewed, manually refined, and fact-checked by the authors to ensure that the final illustration accurately reflects the study’s underlying hypothesis and conforms to scientific standards. Apart from this specified use for Figure 1, no generative AI was employed for data collection, statistical analysis, result interpretation, or manuscript composition.

## 3. Results

### 3.1. Clinical Measures

In this study, all behavioral correlation analyses, GMV, fALFF, and seed-based rsFC analyses were performed on the full sample of 867 participants (414 men). Task-fMRI analyses were conducted on the subset of 687 participants (345 men) who had passed quality control for both resting-state and task-fMRI scans.

To test Hypothesis 1 (depression scores are positively correlated with sleep disturbances and negative emotions), we first examined the behavioral correlates of depression scores. Table 1 shows group differences in demographics and clinical measures; significant threshold was set at *p* < 0.001. There were no sex differences in depression score (Table 1). Significant sex differences were found for age, cognition and emotion scales, e.g., men showed higher fluid intelligence and more anger than women, and women showed higher episodic memory and more fear than men. Based on the results above, sex and age (TIV for GMV analyses) were used as covariates.

Depression score was not related to any cognition scale (all *p*’s > 0.070). Higher depression score was associated with worse sleep quality (r = 0.37, *p* < 0.001) and more pain intensity (r = 0.23, *p* < 0.001), but was not associated with other sensory scales (all *p*’s > 0.011).

Higher depression score was associated with more negative emotion (anger affect: r = 0.47, anger hostility: r = 0.44, anger physical aggression: r = 0.18, fear affect: r = 0.56, fear somatic: r = 0.35, sadness: r = 0.61, loneliness: r = 0.48, perceived hostility: r = 0.30, perceived rejection: r = 0.35, perceived stress: r = 0.56. all *p*’s < 0.001) and less positive emotion (life satisfaction: r = −0.40, mean purpose: r = −0.40, positive affect: r = −0.42, friendship: r = −0.32, emotional support: r = −0.33, instrumental support: r = −0.19, self-efficacy: r = −0.33, all *p*’s < 0.001).

### 3.2. Structural Results

No significant association was found for GMV in whole-brain linear regression against depression score.

### 3.3. Resting-State Functional Connectivity and Falff Results

**Figure 2:**
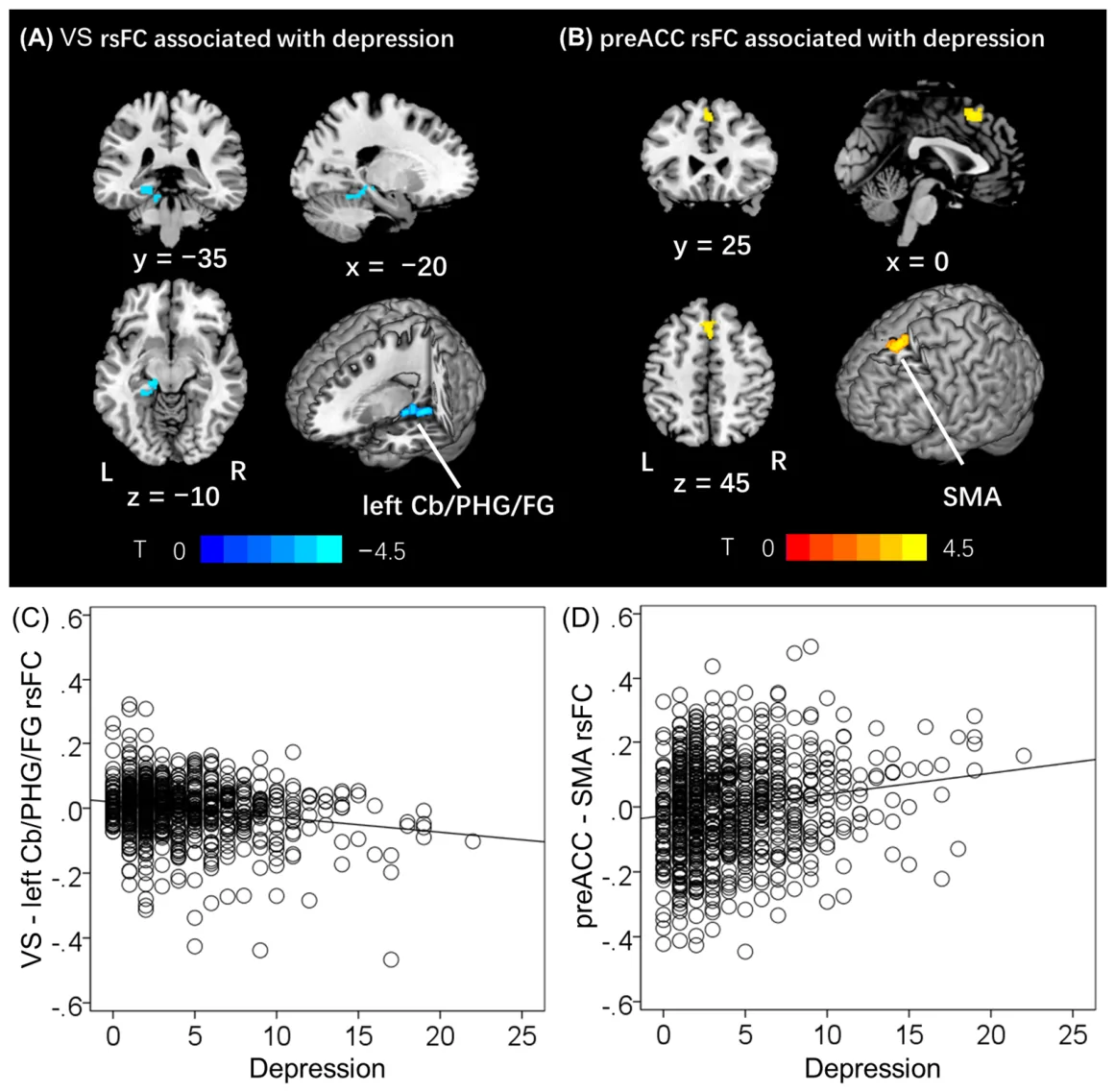
Depression score linearly modulates resting-state functional connectivity (rsFC) of the VS and preACC. (**A**) VS-left Cb/PHG/FG rsFC and (**B**) PreACC-SMA rsFC shows a negative (r = −0.20, *p* < 0.001) and positive (r = 0.16, *p* < 0.001) association with depression score with age and sex as covariates. (**C**,**D**) plotted the scatter plot of VS-left Cb/PHG/FG rsFC and PreACC-SMA rsFC. Threshold: voxel *p* < 0.001, uncorrected combined with cluster *p* < 0.05, FWE corrected. Color bars show voxel t values; warm: positive, cool: negative. L: left, R: right, VS: ventral striatum, periaqueductal gray, PHG: parahippocampal gyrus, FG: fusiform gyrus, Cb: cerebellum, preACC: pregenual anterior cingulate cortex, SMA: supplementary motor area, rsFC: resting state functional connectivity. Note that the residuals are plotted here with age and sex accounted for in all regressions.

To test Hypothesis 2 (in the resting state, depression scores are negatively correlated with rsFC in reward-related pathways and positively correlated with rsFC in cognitive control-related pathways), we performed whole-brain regression analyses for each seed region. The rsFC between VS and a cluster involving the left cerebellum, parahippocampal gyrus and fusiform gyrus (left Cb/PHG/FG, x, y, z = −16, −46, −16; T = −4.56, 1328 mm^3^; Figure 2A) was significantly negatively correlated with depression score (r = −0.20, *p* < 0.001). The rsFC between preACC and supplementary motor area (SMA, x, y, z = 0, 20, 52; T = 4.22, 1120 mm^3^; Figure 2B) was significantly positively correlated with depression score (r = 0.16, *p* < 0.001). We drew the scatter plot for VS-left Cb/PHG/FG rsFC and preACC-SMA rsFC against depression score (Figure 2C,D).

No significant cluster was found for the other seed-based rsFC analyses (hippocampus, BNM, PAG, VTA, hypothalamus, supraACC, subACC, amygdala) or fALFF in whole-brain linear regression against depression score.

The correlation coefficient between VS-left Cb/PHG/FG rsFC and PreACC-SMA rsFC and clinical measures was summarized in Supplementary Table S1. Only VS-left Cb/PHG/FG rsFC showed a significant negative association with PSQI.

In the unrelated-participant rsFC subsample (*n* = 409), the two reported rsFC associations remained directionally consistent with the main analyses: VS-left Cb/PHG/FG rsFC (r = −0.17, *p* < 0.001) and preACC-SMA rsFC (r = 0.17, *p* = 0.001). Similar effect directions were also observed after including BMI, education, race, and substance use as additional covariates (Supplementary Tables S2 and S3).

### 3.4. Task Fmri Results

**Figure 3:**
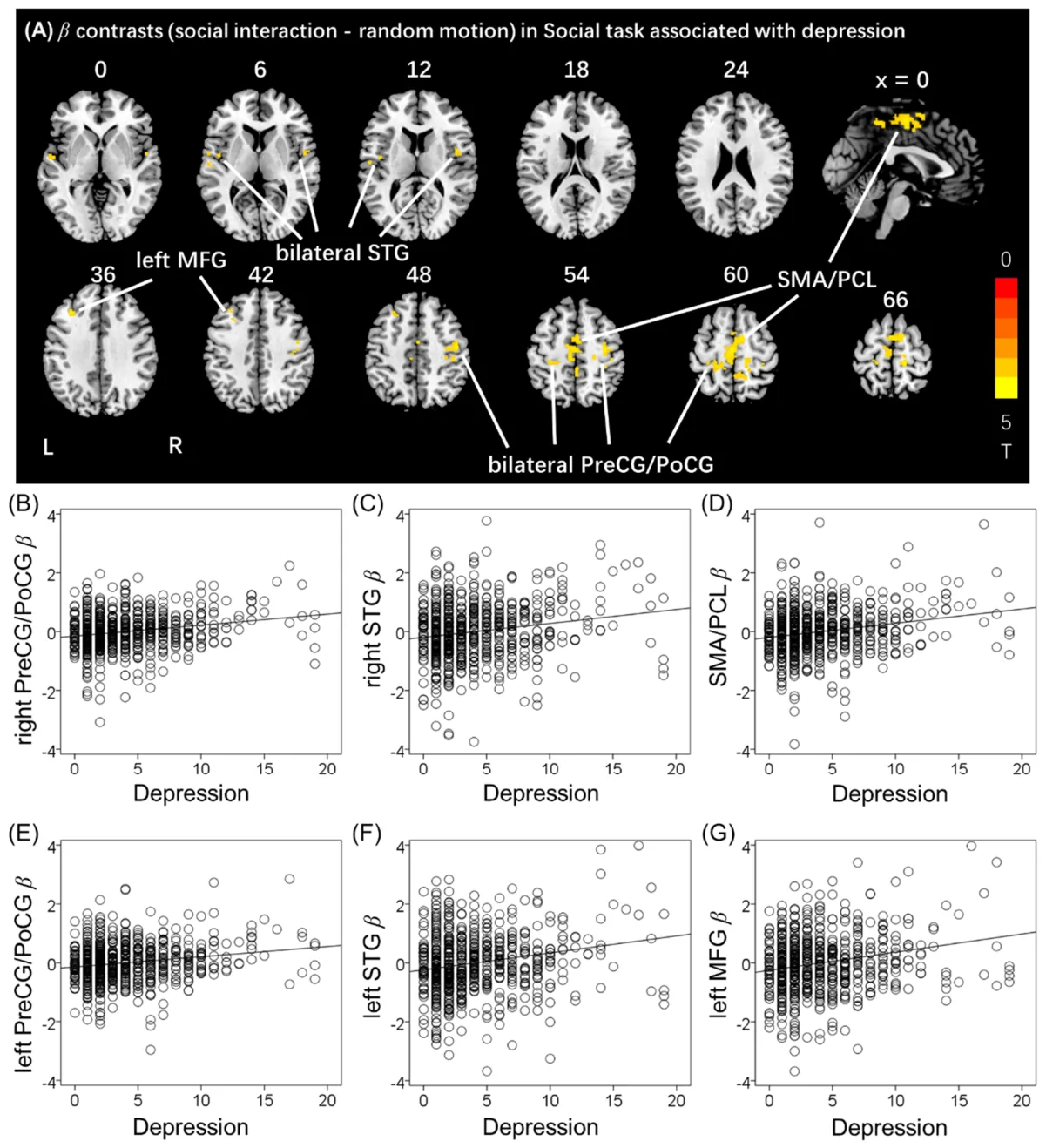
Depression score modulates activation (contrast: social interaction–random motion) of the social cognition task. (**A**) bilateral PreCG/PoCG, bilateral STG, SMA/PCL and left MFG showed positive relationship with depression score. (**B**–**G**) plotted the scatter plot of right PreCG/PoCG (r = 0.21, *p* < 0.001), right STG (r = 0.18, *p* < 0.001), SMA/PCL (r = 0.22, *p* < 0.001), left PreCG/PoCG (r = 0.19, *p* < 0.001), left STG (r = 0.16, *p* < 0.001) and left MFG (r = 0.19, *p* < 0.001) activation against depression score, respectively. Threshold: voxel *p* < 0.001, uncorrected combined with cluster *p* < 0.05, FWE corrected. Color bars show voxel t values, warm: positive. L: left, R: right. Note that the residuals are plotted here with age and sex accounted for in all regressions. PreCG/PoCG: precentral/postcentral gyrus; SMA/PCL: supplementary motor area/paracentral lobule; MFG: middle frontal gyrus; STG: superior temporal gyrus.

To test Hypothesis 3 (among task-fMRI paradigms, social perception may be more sensitive to depression scores than other tasks), we examined the association between depression scores and brain activation across six HCP task paradigms, as well as task performance. For the social perception task, activation (contrast: social interaction–random motion) in the bilateral precentral/postcentral gyrus (PreCG/PoCG; right: r = 0.21, *p* < 0.001; left: r = 0.19, *p* < 0.001; Figure 3A and Table 2), bilateral superior temporal gyrus (STG; right: r = 0.18, *p* < 0.001; left: r = 0.16, *p* < 0.001), SMA/paracentral lobule (PCL; r = 0.22, *p* < 0.001), and left middle frontal gyrus (MFG; r = 0.19, *p* < 0.001) showed a significant positive relationship with depression score. Concurrently, the accuracy rate difference (social interaction–random motion) also showed a positive relationship (r = 0.12, *p* = 0.003) with depression score.

No significant associations with depression score were detected for other contrasts in working memory (2-back–0-back), emotion recognition (negative–neutral), relational reasoning (relational–control), language comprehension (story–math), and gambling (reward–punishment, reward–baseline, punishment–baseline) tasks.

The correlation coefficient between regional activation during the social task and clinical measures was summarized in Table S4. Briefly, social task activation showed significant associations with negative emotion, e.g., fear and sadness scores. In addition, the relationship between social task activation and rsFC measures was not formally tested, as the study was designed to characterize modality-specific associations with depression scores rather than to model cross-modal interactions.

In the unrelated-participant task-fMRI subsample (*n* = 294), the six reported social-task activation associations remained directionally consistent with the main analyses (r = 0.16–0.21, all *p* ≤ 0.006). Similar effect directions were also observed after including BMI, education, race, and substance use as additional covariates (Supplementary Tables S2 and S3).

## 4. Discussion

### 4.1. Summary of Main Findings

**Figure 4:**
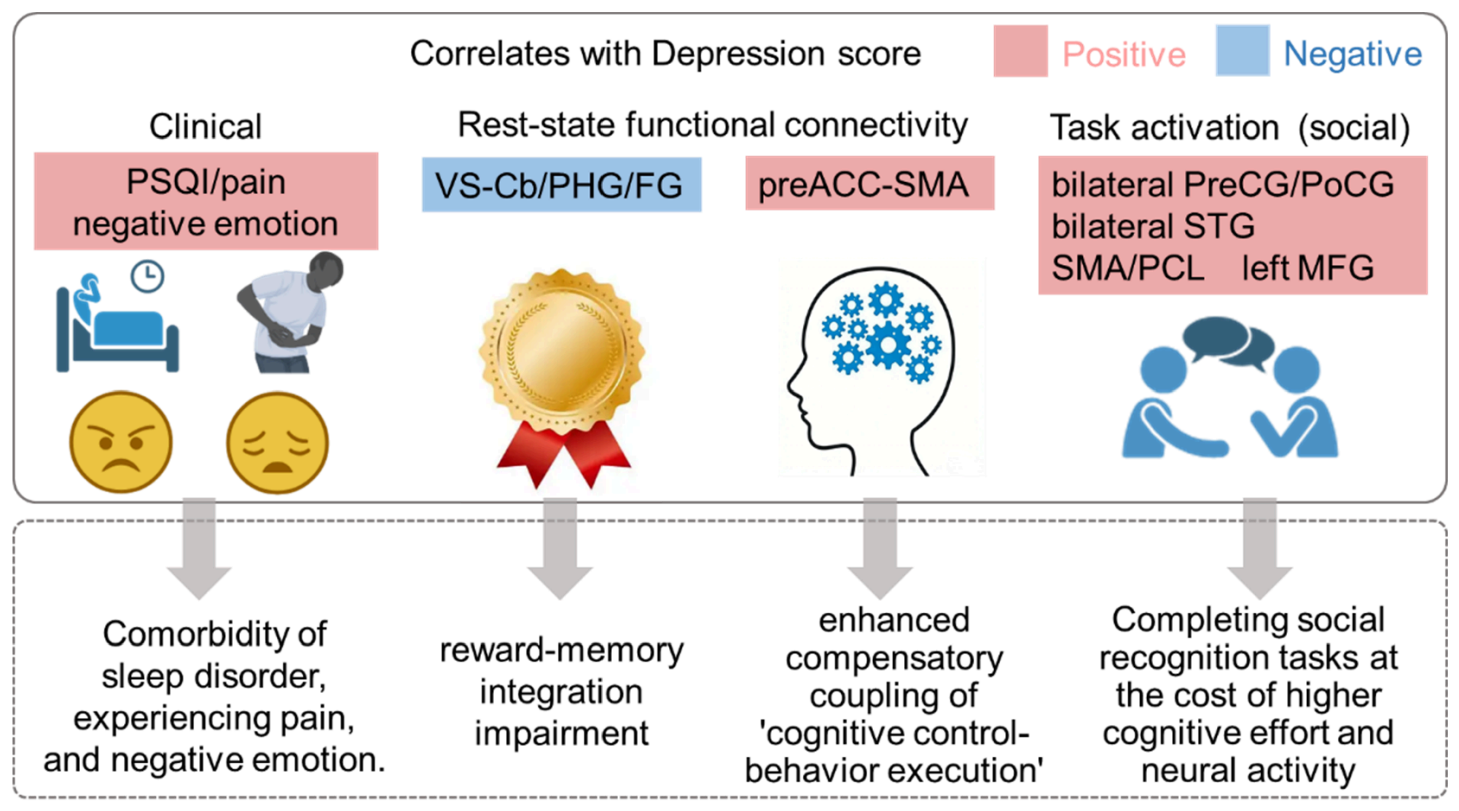
Schematic summary of the main findings and interpretive framework. Solid boxes indicate empirical findings from this study: (1) behavioral: depression scores were positively correlated with sleep disturbances, negative emotions, and pain; (2) rsFC: depression scores were negatively correlated with VS-left Cb/PHG/FG rsFC and positively correlated with preACC-SMA rsFC; (3) task-fMRI: depression scores were positively correlated with social task activation (bilateral PreCG/PoCG, bilateral STG, left MFG, SMA/PCL) and social task accuracy. Solid arrows denote associations observed in the present sample. Dashed boxes represent our interpretive hypotheses—namely, “reward–perception decoupling,” “hyper-connected cognitive control,” and “compensatory hypervigilance”—which are speculative interpretations of the correlational findings rather than empirically demonstrated mechanisms (see discussion). VS: ventral striatum, periaqueductal gray, PHG: parahippocampal gyrus, FG: fusiform gyrus, Cb: cerebellum, preACC: pregenual anterior cingulate cortex, SMA: supplementary motor area, rsFC: resting state functional connectivity. PreCG/PoCG: precentral/postcentral gyrus; SMA/PCL: supplementary motor area/paracentral lobule; MFG: middle frontal gyrus; STG: superior temporal gyrus.

The core findings are summarized below (Figure 4). First, at the behavioral level, depression scores were positively correlated with sleep disturbances (PSQI), negative emotions (anger, fear, etc.), and pain ratings. Second, at the resting-state level, depression scores were negatively correlated with VS-Cb/PHG/FG rsFC, yet positively correlated with preACC-SMA rsFC. Third, at the task-fMRI level, only in the social perception task were positive correlations observed between depression scores and regional activations (bilateral PreCG/PoCG, bilateral STG, left MFG, and SMA/PCL), accompanied by a positive correlation between depression scores and task accuracy.

### 4.2. Behavioral Comorbidity: Depression, Sleep, Negative Emotion, and Pain

This study found that, in a non-clinical sample, depression scores were significantly positively correlated with sleep quality, negative emotions (anger, fear, etc.), and pain scores. This finding is highly consistent with a substantial body of existing literature and further extends these associations to the subclinical dimensional level.

The relationship between depression and sleep disturbances has been extensively documented [32,33]. Moyses-Oliveira et al. found that PSQI scores were significantly correlated with Beck Depression Inventory scores, and the polygenic scores for both measures showed significant overlap, suggesting that sleep quality and depressive symptoms share a genetic basis, with implicated genes converging on common neural pathways [34]. Sleep disturbance significantly mediates the relationship between chronotype and depressive symptoms in young adults [35]. Among university students, poor sleep quality and short sleep duration are significantly associated with a higher prevalence of depressive symptoms [36,37].

Chronic pain is closely related to MDD and anxiety disorders, with high comorbidity rates and shared risk factors. A meta-analysis reviewed how depression can alter pain processing and increase pain sensitivity [38]. The present findings suggest that even in non-clinical populations, elevated depression scores are closely associated with enhanced pain perception, potentially reflecting the possibility that the shared neural substrates of depression and pain—such as thalamic structural and functional alterations [39]—are already operational at the subclinical level.

The relationship between negative emotions and depression also has a robust neurobiological foundation. The amygdala and nucleus accumbens are brain regions closely associated with emotion regulation in humans and play critical roles in depression [40]. Studies have found that depressed patients exhibit negative biases toward negatively valenced emotional stimuli [41]. Both fear and anger, whether excessive or deficient, increase the risk of mental disorders and should be considered in genetic, neurobiological, and neuroimaging studies [42].

In summary, the behavioral finding demonstrates that these comorbidity features—specifically characterized by sleep disturbances, heightened negative emotions, and enhanced pain perception—associated with depression already manifest at the subclinical level, providing a phenotypic foundation for the subsequent neuroimaging findings.

### 4.3. Rsfc: Reward-Perception Decoupling and Hyper-Cognitive Control Coupling

The VS is a core region for reward and motivational processing [43,44]. Individuals with a history of depression exhibit significant alterations in functional connectivity between the VS and regions including the parahippocampal gyrus and inferior temporal gyrus [42]. A recent study found that a history of depression in adolescents was associated with decreased VS-PHG rsFC [45]. The present study further extends this finding to the subclinical dimensional level: VS-Cb/PHG/FG rsFC decreases with increasing depression scores. The PHG and FG are involved in scene construction and visual object/facial recognition, while the cerebellum participates in cognitive prediction and emotional regulation. One possible interpretation is that this pattern represents a form of “reward-perception decoupling”, which could be a network-level correlate of anhedonia. However, alternative explanations are equally plausible—for instance, decreased VS-Cb connectivity could reflect reduced integration of reward signals with cognitive or motor preparation systems, rather than a specific perceptual decoupling. Moreover, because rsFC does not distinguish the direction or excitatory/inhibitory nature of the connections, we cannot determine whether this reduction reflects weaker feed-forward reward signaling or stronger feedback inhibition from visual regions to the VS.

The preACC is a core hub for cognitive control and conflict monitoring, while the SMA is responsible for motor planning and execution. The present study found a positive correlation between depression scores and preACC-SMA rsFC, suggesting that higher depressive tendencies are associated with stronger connectivity between the cognitive “monitoring system” and the “motor system” in the resting state. A meta-analysis reported that MDD patients showed hyperconnectivity between preACC and superior and inferior parietal lobule [46]. Another study found higher preACC-SMA rsFC in MDD patients [47]. The present study found that higher preACC-SMA connectivity is not merely present in clinically depressed patients but is already observable along the continuous dimension of depression scores.

This “cognitive control–motor overcoupling” may reflect excessive rumination or an over-prepared state prior to motor inhibition in depressive traits—that is, the brain remains in a state of high tension characterized by “self-monitoring” and “prepared action” even in the resting state. This overcoupling may reflect a misallocation of cognitive control resources toward the motor preparation system, and this “mismatch” of neural resources may be the neural basis for the rigidity or slowness observed in depressed individuals when they need to flexibly switch cognitive states. Nevertheless, alternative interpretations should be considered. For example, this increased connectivity could simply reflect a general state of heightened arousal or anxiety, which often co-occurs with depressive symptoms, rather than a depression-specific cognitive control aberration. It is also possible that this connectivity pattern represents an adaptive upregulation of cognitive control to compensate for perceived difficulty in regulating emotions, rather than a maladaptive “overcoupling”.

### 4.4. Social Task: The Compensatory Hypervigilance Hypothesis

Among the several task-fMRI paradigms, only the social task was specifically associated with depression scores. One of the core features of depression is precisely impairment in interpersonal functioning and biases in social cognition [1]. Therefore, the fact that only social task showed significant results may be because it is most directly related to the core social dysfunction domain of depression—namely, the ways in which social information is encoded, interpreted, and responded to.

PreCG/PoCG are involved in action observation, imitation, and somatosensory representation. STG is a key region for language comprehension and social perception. SMA involved in cognitive control and motor planning, potentially reflecting conscious monitoring of social rules and response preparation in social contexts. A previous meta-analysis found that MDD patients exhibited hyperactivation in bilateral superior/middle frontal gyri during social processing tasks [1]. In a reasoning task study, the subclinical depressed group exhibited greater superior parietal/prefrontal cortex activation during both learning and test phases, which represented a compensatory mechanism to achieve the same behavioral performance as the non-depressed group [48]. A recent meta-analysis also reported that individuals with subclinical depression exhibit increased regional activation during emotion, reward, and decision-making tasks [49].

In socio-affective processing, depressed patients show enhanced neural responses to social feedback. A study of depressed women found that the depressed group exhibited emotional and neural hyperresponsivity to both romantic social rejection and acceptance [50]. Another study of female adolescents at risk for depression found that high-risk individuals exhibited heightened neural activity and functional connectivity responses following social rejection [51]. In social comparison tasks, patients with depression and social anxiety showed greater default mode network activation during upward social comparisons [52]. These findings are consistent with the present study—individuals with higher depressive tendencies indeed mobilize greater neural resources when confronted with socially relevant information.

The depression scores were also positively correlated with task accuracy. In socio-affective processing, depressed patients showed enhanced amygdala responses to both positive and negative social feedback [53]. In social exclusion paradigms, adolescents at risk of depression exhibited atypical recruitment of emotional salience and regulation networks during social exclusion [54]. In non-clinical samples, individuals with higher depression scores do not simply exhibit “hypofunction” in social cognitive tasks; rather, they mobilize greater neural resources for “over-encoding” and “analytical processing.” They are not “naturally perceiving” social information but rather “effortfully computing” social rules and intentions. This additional cognitive investment may stem from hypervigilance toward potential threats in social contexts (such as rejection, criticism, or misunderstanding). While this hypervigilance consumes more neural resources, it unexpectedly yields improved behavioral performance—i.e., higher accuracy. In more severe clinical depression, this compensatory mechanism may fail, and instead manifest as a comprehensive impairment of social function. Alternative explanations should also be considered: (1) the enhanced activation could reflect greater task engagement or motivation in higher-scoring individuals rather than a “compensatory” mechanism per se; (2) the positive correlation with accuracy might be driven by the specific nature of the HCP social task (identifying social vs. random motion), which may not directly generalize to more complex social cognitive processes such as mentalizing or empathy; and (3) it is also possible that this pattern is specific to the non-clinical range of depression scores and may not extrapolate to clinical depression, where such compensatory mechanisms may fail and manifest as social function impairment.

### 4.5. Interpretation of Null Findings

This study found no significant associations between depression scores and GMV, fALFF, other seed-based rsFCs or task-fMRI paradigms. The null results for GMV and fALFF suggest that, at the subclinical stage, depressive symptoms may manifest primarily as functional integration deficits across brain networks (i.e., “disconnection” at the network level) rather than as structural atrophy or altered spontaneous activity in local regions. The absence of findings in other tasks does not statistically demonstrate that social cognition is more sensitive to depressive symptoms than other cognitive domains. We therefore interpret this finding tentatively: social cognitive processing may be a particularly relevant domain to examine in relation to dimensional depressive symptoms, and the null findings in other tasks should be interpreted with caution, as they may reflect task design characteristics, statistical power limitations, or the specific nature of the non-clinical sample.

### 4.6. Limitations

This study has several limitations. First, the sample was drawn from the healthy adult population of the HCP, with depression scores overall in the subclinical range; generalization of the findings to clinically depressed patients requires caution. Second, this study employed a cross-sectional design and cannot infer causality—that is, whether the observed brain connectivity abnormalities are causes or consequences of depressive tendencies remains unclear. Third, the social task design in the HCP may not fully cover all dimensions of social cognition (such as empathy, theory of mind, etc.). Fourth, our study involved multiple analytical streams, and we did not apply a single unified correction across all analyses. While cluster-level FWE correction was applied within each brain map, this does not control the family-wise error rate for the entire study—a major limitation of multimodal exploratory work. All findings should therefore be considered hypothesis-generating and require independent replication in pre-registered studies. Lastly, we did not perform formal cross-task statistical comparisons (e.g., task × depression score interaction) to test whether social perception is significantly more sensitive to depressive symptoms than other domains. Our inference is based solely on the observation that only the social task yielded significant results in our sample. Direct comparative analyses in future studies are needed to confirm the specificity of this association.

### 4.7. Conclusions

This multimodal HCP-based study demonstrates that dimensional depressive symptoms are associated with distinct patterns of rsFC and social-task-evoked neural responses in non-clinical populations. These findings support a dimensional perspective on depression and provide preliminary neuroimaging evidence for subclinical symptom manifestations. However, given the correlational design, these interpretations require independent replication and causal validation through future longitudinal or experimental studies.

## Figures and Tables

**Table 1:** Demographics and clinical measures of the participants.

Characteristic	Men (*n* = 414)	Women (*n* = 453)	*t*	*p*
**Demographics**				
Age, years	27.9 ± 3.6	29.5 ± 3.6	−6.60	<0.001
Race (White/AA/AH/AI/More/Unknown)	327/44/25/1/10/7	341/62/27/1/12/104	--	--
Ethnicity (Latino/non-Latino/Unknown)	49/360/5	35/413/5	--	--
Education, years	14.9 ± 1.7	15.0 ± 1.8	−0.77	0.443
BMI	26.8 ± 4.3	26.0 ± 5.8	2.17	0.031
Income	5.1 ± 2.2	5.1 ± 2.2	−0.32	0.752
PSQI	4.6 ± 2.5	4.9 ± 3.1	−1.83	0.067
Depression	3.9 ± 3.5	4.2 ± 3.6	−1.07	0.287
**Cognition Scales**				
Episodic Memory (PicSeq)	110.2 ± 13.4	113.6 ± 12.7	−3.85	<0.001
Cognitive Flexibility (CardSort)	116.4 ± 10.2	114.7 ± 10.2	2.46	0.014
Inhibition (Flanker)	113.4 ± 10.7	110.4 ± 9.2	4.28	<0.001
Fluid Intelligence (PMAT24_A_CR)	17.9 ± 4.5	16.5 ± 4.7	4.36	<0.001
Reading Decoding (ReadEng)	118.7 ± 10.8	116 ± 10.3	3.76	<0.001
Vocabulary Comprehension (PicVocab)	118.3 ± 9.5	116.3 ± 9.1	3.29	0.001
Processing Speed (ProcSpeed)	115.9 ± 16.3	115.2 ± 14.8	0.62	0.534
Impulsivity (DDisc_AUC_200)	0.3 ± 0.2	0.2 ± 0.2	1.81	0.070
Impulsivity (DDisc_AUC_40K)	0.5 ± 0.3	0.5 ± 0.3	0.75	0.455
Spatial Orientation (VSPLOT_TC)	16.1 ± 4.2	14.2 ± 4.4	6.76	<0.001
Sustained Attention (SCPT_SEN)	1.0 ± 0.1	0.9 ± 0.1	1.70	0.089
Verbal Episodic Memory (IWRD_TOT)	35.4 ± 2.9	35.9 ± 2.9	−2.68	0.008
Working Memory (ListSort)	112.3 ± 11	110.5 ± 11.5	2.30	0.022
**Emotion Scales**				
Emotion Recognition (ER40_CR)	35.5 ± 2.6	35.8 ± 2.5	−1.79	0.073
Anger Affect	47.9 ± 8.5	47.6 ± 8.0	0.63	0.529
Anger Hostility	51.1 ± 8.6	50 ± 8.4	1.82	0.070
Anger Physical Aggression	54.1 ± 9.2	49.7 ± 7.8	7.54	<0.001
Fear Affect	49.0 ± 7.9	51.3 ± 7.9	−4.13	<0.001
Fear Somatic	51.4 ± 8.0	52.2 ± 8.4	−1.41	0.160
Sadness	45.9 ± 8.0	46.4 ± 8.0	−0.97	0.332
Life Satisfaction	54.3 ± 8.6	54.8 ± 9.4	−0.80	0.425
Mean Purpose	51.1 ± 8.7	52.5 ± 8.5	−2.48	0.013
Positive Affect	49.9 ± 7.8	50.4 ± 7.8	−0.88	0.381
Friendship	50.5 ± 8.8	50.4 ± 9.4	0.03	0.974
Loneliness	51.0 ± 9.1	51.2 ± 8.1	−0.25	0.806
Perceived Hostility	49.8 ± 8.4	47.6 ± 8.7	3.68	<0.001
Perceived Rejection	48.6 ± 8.9	48.3 ± 8.5	0.54	0.591
Emotional Support	50.3 ± 10.0	52.5 ± 9.2	−3.36	0.001
Instrumental Support	47.9 ± 9.5	48.1 ± 8.6	−0.44	0.657
Perceived Stress	47.6 ± 9.2	48.7 ± 9.0	−1.83	0.068
Self-efficacy	52.2 ± 8.5	50.2 ± 8.0	3.57	<0.001
**Sensory Scales**				
Audition (words in noise)	4.4 ± 1.5	4.3 ± 1.4	0.81	0.418
Olfaction (odor identification test)	109.5 ± 9.1	111.7 ± 9.0	−3.63	<0.001
Pain (pain intensity surveys)	1.3 ± 1.5	1.4 ± 1.8	−0.57	0.566
Taste (taste intensity test)	92.4 ± 14.1	97.6 ± 13.9	−5.48	<0.001
Vision (EVA scores & farnsworth test)	15.0 ± 5.0	15.1 ± 3.9	−0.51	0.609
Mars contrast sensitivity	1.9 ± 0.9	1.8 ± 0.1	1.45	0.147

Note: Values are mean ± SD. AA: African American or Black. AH: Asian or Native Hawaiian. AI: Native Alaskan or Indian American. More: more than one race. Unknown: unknown or not reported. Latino: Hispanic/Latino. Non-Latino: not Hispanic/Latino. Total household income: <$10,000 = 1, 10K–19,999 = 2, 20K–29,999 = 3, 30K–39,999 = 4, 40K–49,999 = 5, 50K–74,999 = 6, 75K–99,999 = 7, ≥100,000 = 8.

**Table 2:** Clusters of positive activation (social interaction–random motion) during social task in whole-brain linear regression against depression score.

Region	Cluster Size (k)	Peak Voxel (T)	Cluster FWE *p*	MNI Coordinate (mm)		
				X	Y	Z
right PreCG/PoCG	251	5.04	0.000	34	−16	52
SMA/PCL	917	4.97	0.000	−2	−16	58
left MFG	104	4.65	0.005	−32	28	36
left PreCG/PoCG	191	4.63	0.000	−18	−36	58
right STG	70	4.28	0.033	52	−4	2
left STG	115	3.79	0.003	−56	−14	10

Note: PreCG/PoCG: precentral/postcentral gyrus; SMA/PCL: supplementary motor area/paracentral lobule; MFG: middle frontal gyrus; STG: superior temporal gyrus.

## Data Availability

All data needed to evaluate the conclusions in the paper are shown in the paper and the supplement. All raw data supporting the analyses and findings of this study are available from the Human Connectome Project, at https://www.humanconnectome.org/. accessed on 9 October 2019.

## Supplementary Materials

The following supporting information can be downloaded at: https://www.mdpi.com/article/10.3390/brainsci16080884/s1, Table S1: The correlation coefficient between VS—left Cb/PHG/FG rsFC and PreACC—SMA rsFC and clinical measures with age and sex as covariates; Table S2: The sensitivity analyses results of unrelated participants (unique family IDs, i.e., only one participant per family); Table S3: The sensitivity analyses results of additional covariates (BMI, years of education, race, substance use), Table S4: The correlation coefficient between regional activation during social task and clinical measures with age and sex as covariates.
