# Supplementary material for: Association of Depressive Symptom Scores with Multimodal Brain Imaging and Behavioral Phenotypes: A Resting-State, Task-FMRI, and Clinical Comorbidity Study Based on the Human Connectome Project
Source: Brain Sci. 2026 Aug 19;16(8):884. doi: 10.3390/brainsci16080884 (PMC13510362; doi:10.3390/brainsci16080884)
Supplement: Supplementary file 1 [file brainsci-16-00884-s001.zip › brainsci-4458020-supplementary.pdf]

Supplement:

Association of depressive symptom scores with multimodal brain imaging and behavioral phenotypes: a resting-state, task-fMRI, and clinical comorbidity study based on the Human Connectome Project

Supplementary Methods

Imaging protocol and data preprocessing

MRI was done using a customized 3 T Siemens Connectome Skyra with a standard 32-channel Siemens receiver head coil and a body transmission coil. T1-weighted high-resolution structural images were acquired using a 3D MPRAGE sequence with 0.7 mm isotropic resolution (FOV = 224 × 224 mm, matrix = 320 × 320, 256 sagittal slices, TR = 2400 ms, TE = 2.14 ms, TI = 1000 ms, FA = 8°) and used to register functional MRI data to a standard brain space. FMRI data were collected using gradient-echo echo-planar imaging (EPI) with 2.0 mm isotropic resolution (FOV = 208 × 180 mm, matrix = 104 × 90, 72 slices, TR = 720 ms, TE = 33.1 ms, FA = 52°, multi-band factor = 8).

Participants completed 7 task and 2 resting-state (rs) scans on two separate days. Within each rs-fMRI scan, oblique axial acquisitions alternated between phase encoding in a right-to-left (RL) direction in one run and left-to-right (LR) direction in the other run. Each run lasted 14.4 min (1200 frames). Cardiac and respiratory data were also acquired, using a standard Siemens pulse oximeter placed on a digit and a respiratory belt placed on the abdomen. These physiological signals were sampled at 400 Hz (~288 samples per frame). More details of the data collection procedures can be found in the HCP S1200 Release Reference Manual.

In the current study, only the data of the first rs-fMRI session (two runs: LR and RL) were used and processed with Statistical Parametric Mapping (SPM8, Wellcome Department of Imaging Neuroscience, University College London, U.K.). Images of each participant were first realigned (motion corrected) and a mean functional image volume was constructed from the realigned image volumes. These mean images were co-registered with the high-resolution structural MPRAGE image and segmented for normalization with affine registration followed by nonlinear transformation. The normalization parameters determined for the structural volume were then applied to the corresponding functional volumes for each participant. Afterwards, the images were smoothed with a Gaussian kernel of 4 mm at Full Width at Half Maximum.

White matter and cerebrospinal fluid signals, whole-brain mean signal, and physiological signals were regressed out to reduce spurious BOLD variances and to eliminate cardiac- and respiratory artifacts. A temporal band-pass filter (0.009 Hz < f < 0.08 Hz) was applied to the time course to obtain low-frequency fluctuations, as in our previous studies [1]. Lastly, we used "scrubbing" to further eliminate global motion-related artifacts. Specifically, frame-wise displacement given by $FD(t) = |\Delta d_x(t)| + |\Delta d_y(t)| + |\Delta d_z(t)| + |\Delta\alpha(t)| + |\Delta\beta(t)| + |\Delta\gamma(t)|$ was

computed for every time point t, where (d_x, d_y, d_z) and (α, β, γ) were the translational and rotational movements, respectively [2]. Moreover, the root mean square variance of the differences (DVARS) in % BOLD intensity $I(t)$ between consecutive time points across brain voxels, was computed as: $DVARS(t)$ = sqrt($|I(t) - I(t\text{-}1)|^2$), where the brackets indicate the mean across brain voxels. Following previous HCP studies, we marked volumes with FD > 0.2 mm or DVARS > 75 as well as one frame before and two frames after these volumes as outliers (censored frames). Uncensored segments of data lasting fewer than five contiguous volumes were also labeled as censored [3]. A total of 146 participants with more than half of the frames censored in either run was removed from further analyses. The two runs were combined before computing the resting state functional connectivity.

Voxel-based morphometry

VBM analysis identifies differences in the local composition of brain tissue, accounting for large-scale variation in gross anatomy and location. The analysis includes spatially normalizing individuals' structural images to the same stereotactic space, segmenting the normalized images into distinct brain tissues, and smoothing the gray matter images. We used the raw images to avoid potential interference with the CAT12 preprocessing pipeline. T1-images were first co-registered to the MNI template using a multiple-stage affine transformation during which the 12 parameters were estimated. Co-registration was performed with a coarse affine registration using mean square differences, followed by a fine affine registration using mutual information. Coefficients of the basis functions that minimized the residual squared difference between individual image and the template were estimated. Tissue probability maps constructed from 452 healthy subjects were used in affine transformation, and affine regularization was performed with ICBM space template. T1 images were then corrected for intensity bias field and a local means denoising filter and segmented into cerebrospinal fluid, gray, and white matter. Segmented and the initially registered tissue class maps were normalized using DARTEL [4] a fast diffeomorphic image registration algorithm of SPM. As a high-dimensional non-linear spatial normalization method, DARTEL generates mathematically consistent inverse spatial transformations. We used the standard DARTEL template in MNI space, constructed from 555 healthy subjects of the IXI-database (http://www.brain-development.org/, accessed on 8 August 2026), to drive the DARTEL normalization. Skull-stripping and final clean up (to remove remaining meninges and correct for volume effects in some regions) were performed with default parameters. Normalized GM maps were modulated to obtain the absolute volume of GM tissue corrected for individual brain sizes. Finally, the GM maps were smoothed by convolving with an isotropic Gaussian kernel (FWHM = 8mm).

For the regions of interest (ROIs) identified from group analyses, we used MarsBar (http://marsbar.sourceforge.net/, accessed on 8 August 2026) to derive for individual subjects the β estimates of the brain features.

Supplementary Results

Supplementary Table S1. The correlation coefficient between VS - left Cb/PHG/FG rsFC and PreACC - SMA rsFC and clinical measures with age and sex as covariates.

Characteristic		VS - left Cb/PHG/FG	PreACC - SMA
Depression	r	-.20	.16
	p	.000	.000
PSQI	r	-.13	.06
	p	.000	.099
**Cognition scales**			
Episodic Memory (PicSeq)	r	.08	.01
	p	.015	.714
Cognitive Flexibility (CardSort)	r	.03	-.01
	p	.362	.695
Inhibition (Flanker)	r	.01	-.00
	p	.757	.976
Fluid Intelligence (PMAT24_A_CR)	r	.02	.03
	p	.629	.436
Reading Decoding (ReadEng)	r	-.01	-.01
	p	.771	.826
Vocabulary Comprehension (PicVocab)	r	-.02	-.02
	p	.655	.553
Processing Speed (ProcSpeed)	r	-.01	-.02
	p	.890	.637
Impulsivity (DDisc_AUC_200)	r	-.03	-.07
	p	.352	.057
Impulsivity (DDisc_AUC_40K)	r	-.04	-.07
	p	.263	.053
Spatial Orientation (VSPLOT_TC)	r	.06	.03
	p	.081	.424
Sustained Attention (SCPT_SEN)	r	-.04	.03
	p	.279	.348
Verbal Episodic Memory (IWRD_TOT)	r	-.05	-.03
	p	.166	.462
Working Memory (ListSort)	r	-.02	.03
	p	.596	.450
**Emotion scales**			
Emotion Recognition (ER40_CR)	r	.04	.00
	p	.311	.907
Anger Affect	r	.02	-.00
	p	.566	.954
Anger Hostility	r	-.01	.06
	p	.727	.059
Anger Physical Aggression	r	.07	-.05
	p	.033	.148
Fear Affect	r	-.07	.05
	p	.046	.142
Fear Somatic	r	-.09	.02
	p	.006	.530
Sadness	r	-.10	.04
	p	.003	.194
Life Satisfaction	r	-.09	.07
	p	.006	.040
Mean Purpose	r	-.10	.03
	p	.003	.427
Positive Affect	r	-.10	.07
	p	.005	.059

Friendship	r	.11	-.07
	p	.001	.040
Loneliness	r	.11	-.09
	p	.001	.013
Perceived Hostility	r	.06	-.07
	p	.106	.046
Perceived Rejection	r	.08	-.06
	p	.015	.070
Emotional Support	r	-.08	.09
	p	.029	.010
Instrumental Support	r	-.03	.05
	p	.454	.164
Perceived Stress	r	-.03	.09
	p	.329	.011
Self Efficacy	r	.05	-.10
	p	.137	.005
**Sensory scales**			
Audition (words in noise)	r	-.01	.02
	p	.755	.549
Olfaction (odor identification test)	r	.04	.03
	p	.292	.374
Pain (pain intensity surveys)	r	-.08	.04
	p	.027	.304
Taste (taste intensity test)	r	-.01	.05
	p	.814	.143
Vision (EVA scores & farnsworth test)	r	-.07	.01
	p	.033	.708
Mars contrast sensitivity	r	-.05	.05
	p	.192	.121

Note: VS: ventral striatum, periaqueductal gray, PHG: parahippocampal gyrus, FG: fusiform gyrus, Cb: cerebellum, preACC: pregenual anterior cingulate cortex, SMA: supplementary motor area, rsFC: resting state functional connectivity.

Supplementary Table S2. The sensitivity analyses results of unrelated participants (unique family IDs, i.e., only one participant per family).

Characteristic		VS - left Cb/PHG/FG	PreACC - SMA
Main findings (n=867)	r	-.20	.16
	p	.000	.000
Unrelated subsample (n=409)	r	-.17	.17
	p	.000	.001

Characteristic		right PreCG/ PoCG	SMA/ PCL	left MFG	left PreCG/ PoCG	right STG	left STG
**Depression and sleep quality**							
Main findings (n=687)	r	.21	.22	.19	.19	.18	.16
	p	.000	.000	.000	.000	.000	.000
Unrelated subsample (n=294)	r	.19	.21	.19	.18	.18	.16
	p	.001	.000	.002	.002	.002	.006

Note: VS: ventral striatum, periaqueductal gray, PHG: parahippocampal gyrus, FG: fusiform gyrus, Cb: cerebellum, preACC: pregenual anterior cingulate cortex, SMA: supplementary motor area, rsFC: resting state functional connectivity. PreCG/PoCG: precentral/postcentral gyrus; SMA/PCL:

supplementary motor area/paracentral lobule; MFG: middle frontal gyrus; STG: superior temporal gyrus.

Supplementary Table S3. The sensitivity analyses results of additional covariates (BMI, years of education, race, substance use).

Characteristic		VS - left Cb/PHG/FG	PreACC - SMA
Main findings (n=867, age and sex)	r	-.20	.16
	p	.000	.000
Additional covariates added	r	-.20	.15
	p	.000	.000

Characteristic		right PreCG/ PoCG	SMA/ PCL	left MFG	left PreCG/ PoCG	right STG	left STG
**Depression and sleep quality**							
Main findings (n=687)	r	.21	.22	.19	.19	.18	.16
	p	.000	.000	.000	.000	.000	.000
Additional covariates added	r	.21	.23	.19	.18	.19	.17
	p	.000	.000	.000	.000	.000	.000

Note: VS: ventral striatum, periaqueductal gray, PHG: parahippocampal gyrus, FG: fusiform gyrus, Cb: cerebellum, preACC: pregenual anterior cingulate cortex, SMA: supplementary motor area, rsFC: resting state functional connectivity. PreCG/PoCG: precentral/postcentral gyrus; SMA/PCL: supplementary motor area/paracentral lobule; MFG: middle frontal gyrus; STG: superior temporal gyrus. Substance use include: SSAGA_Alc_12_Frq (Alcohol, the sign was reversed to accurately represent the drinking severity), SSAGA_TB_Smoking_History, SSAGA_Times_Used_Cocaine, SSAGA_Times_Used_Hallucinogens, SSAGA_Times_Used_Opiates, SSAGA_Times_Used_Sedatives,SSAGA_Times_Used_Stimulants, and SSAGA_Mj_Times_Used (Marijuana).

Supplementary Table S4. The correlation coefficient between regional activation during social task and clinical measures with age and sex as covariates.

Characteristic		right PreCG/ PoCG	SMA/ PCL	left MFG	left PreCG/ PoCG	right STG	left STG
**Depression and sleep quality**							
Depression	r	.21	.22	.19	.19	.18	.16
	p	.000	.000	.000	.000	.000	.000
PSQI	r	.07	.07	.10	.06	.05	.07
	p	.057	.073	.011	.106	.239	.080
**Cognition scales**							
Episodic Memory (PicSeq)	r	.03	.06	.09	.03	.05	.07
	p	.428	.134	.015	.514	.237	.053
Cognitive Flexibility (CardSort)	r	-.00	-.04	.01	-.01	.04	.02
	p	.952	.279	.849	.823	.276	.646
Inhibition (Flanker)	r	.02	.00	.03	.01	.06	-.01
	p	.653	.961	.521	.708	.137	.826
Fluid Intelligence (PMAT24_A_CR)	r	.03	.04	.04	.01	.06	.05
	p	.372	.324	.345	.732	.100	.239
Reading Decoding (ReadEng)	r	-.01	-.03	-.00	-.00	.02	-.01

	p	.775	.391	.908	.960	.643	.764
Vocabulary Comprehension (PicVocab)	r	.01	-.01	-.01	-.03	.04	.01
	p	.838	.810	.759	.417	.364	.811
Processing Speed (ProcSpeed)	r	-.03	-.04	-.04	-.00	.00	.01
	p	.522	.293	.298	.950	.996	.844
Impulsivity (DDisc_AUC_200)	r	.05	.02	-.01	.01	.05	.03
	p	.227	.622	.732	.716	.238	.474
Impulsivity (DDisc_AUC_40K)	r	.00	-.03	-.05	.01	.04	.00
	p	.969	.500	.161	.714	.260	.987
Spatial Orientation (VSPLOT_TC)	r	-.00	-.07	-.01	-.02	.01	-.03
	p	.921	.080	.854	.534	.900	.408
Sustained Attention (SCPT_SEN)	r	-.01	-.01	-.02	-.01	.02	-.01
	p	.825	.859	.654	.790	.532	.709
Verbal Episodic Memory (IWRD_TOT)	r	-.09	-.06	.02	-.06	-.07	-.05
	p	.021	.095	.640	.119	.065	.210
Working Memory (ListSort)	r	.03	.06	.09	.03	.05	.07
	p	.428	.134	.015	.514	.237	.053
**Emotion scales**							
Emotion Recognition (ER40_CR)	r	.01	-.02	.01	-.06	-.04	-.07
	p	.771	.700	.820	.134	.367	.084
Anger Affect	r	.05	.07	.09	.04	.06	.03
	p	.194	.062	.018	.352	.100	.437
Anger Hostility	r	.04	.07	.13	.06	.07	.08
	p	.322	.067	.000	.124	.063	.045
Anger Physical Aggression	r	-.02	-.06	.01	-.04	-.08	-.07
	p	.591	.108	.792	.284	.035	.091
Fear Affect	r	.12	.14	.14	.09	.13	.10
	p	.001	.000	.000	.017	.001	.008
Fear Somatic	r	.03	.09	.04	.08	.09	.08
	p	.404	.018	.276	.030	.025	.037
Sadness	r	.14	.15	.15	.10	.11	.10
	p	.000	.000	.000	.008	.004	.009
Life Satisfaction	r	-.05	-.01	-.07	.02	.00	-.01
	p	.220	.743	.073	.563	.957	.832
Mean Purpose	r	-.10	-.06	-.12	-.02	-.07	-.04
	p	.013	.153	.002	.627	.087	.290
Positive Affect	r	-.09	-.08	-.13	-.03	-.07	-.04
	p	.023	.033	.001	.491	.060	.296
Friendship	r	-.07	-.05	-.10	-.05	-.05	-.09
	p	.059	.167	.010	.216	.177	.019
Loneliness	r	.02	.06	.10	.04	.04	.05
	p	.529	.117	.010	.261	.350	.225
Perceived Hostility	r	.06	.07	.03	.04	.06	.02
	p	.117	.059	.403	.321	.130	.585
Perceived Rejection	r	.03	.06	.04	.03	.01	.01
	p	.511	.096	.264	.436	.711	.794
Emotional Support	r	-.05	-.06	-.06	-.05	-.08	-.07
	p	.223	.126	.123	.166	.038	.092
Instrumental Support	r	-.05	-.00	-.02	-.02	-.03	-.04
	p	.230	.919	.683	.641	.383	.284
Perceived Stress	r	.09	.09	.12	.07	.08	.07
	p	.024	.014	.002	.057	.029	.068
Self Efficacy	r	-.05	-.04	-.06	-.00	-.05	-.02
	p	.183	.281	.125	.984	.224	.682
**Sensory scales**							
Audition (words in noise)	r	-.02	.02	.04	.03	-.02	.02
	p	.634	.602	.276	.389	.683	.542
Olfaction (odor identification test)	r	.02	.04	-.01	.04	.04	.01
	p	.549	.302	.879	.302	.326	.735

Pain (pain intensity surveys)	r	.03	.04	.08	.00	-.02	.02
	p	.451	.249	.033	.964	.649	.633
Taste (taste intensity test)	r	-.03	.03	.03	.01	-.02	-.01
	p	.384	.434	.442	.721	.621	.827
Vision (EVA scores & farnsworth test)	r	-.01	.00	-.06	.01	-.05	-.04
	p	.838	.990	.108	.874	.218	.331
Mars contrast sensitivity	r	.02	.04	.18	.03	.03	.15
	p	.586	.316	.000	.497	.496	.000

Note: PreCG/PoCG: precentral/postcentral gyrus; SMA/PCL: supplementary motor area/paracentral lobule; MFG: middle frontal gyrus; STG: superior temporal gyrus.

Supplementary References

1. Zhang, S. and C.R. Li, *Ventral striatal dysfunction in cocaine dependence - difference mapping for subregional resting state functional connectivity.* Transl Psychiatry, 2018. **8**(1): p. 119.
2. Power, J.D., et al., *Spurious but systematic correlations in functional connectivity MRI networks arise from subject motion.* Neuroimage, 2012. **59**(3): p. 2142-54.
3. Li, J., et al., *Global signal regression strengthens association between resting-state functional connectivity and behavior.* Neuroimage, 2019. **196**: p. 126-141.
4. Ashburner, J., *A fast diffeomorphic image registration algorithm.* NeuroImage, 2007. **38**(1): p. 95-113.
